# A dilute sodium hydroxide technique for radiolarian extraction from cherts

**DOI:** 10.1038/s41598-024-63755-9

**Published:** 2024-06-17

**Authors:** Tetsuji Onoue, Sakiko Hori, Yuki Tomimatsu, Manuel Rigo

**Affiliations:** 1 Department of Earth and Planetary Sciences, Kyushu University, Fukuoka, 819-0395 Japan; 2 Department of Earth System Science, Fukuoka University, Fukuoka, 814-0180 Japan; 3 Department of Geosciences, University of Padova, 35131 Padova, Italy; 4 IGG-CNR, 35131 Padova, Italy

**Keywords:** Chert, Radiolarians, Sodium hydroxide, Hydrofluoric acid, Triassic, Palaeontology, Stratigraphy, Geochemistry

## Abstract

Radiolarians have been used to determine geological ages and have contributed markedly to our understanding of Earth’s history. Hydrofluoric acid (HF) has traditionally been used to extract radiolarian fossils from siliceous deposits (i.e., radiolarian cherts), but this acid is strictly regulated because of environmental and human health concerns. Here we report on the successful extraction of radiolarians from cherts using a low-concentration NaOH solution (1 mol/L NaOH) as an alternative to HF. The degree of chert dissolution in NaOH is strongly temperature-dependent and is limited at < 80 °C. However, even a 1 mol/L NaOH solution is sufficient to dissolve chert at 100 °C. Our new NaOH method yields better-preserved radiolarian fossils compared with the conventional HF method. The 1 mol/L NaOH solution is less hazardous, easier to handle, and has fewer effects on the environment and human health than HF. Therefore, this method can be widely used for research and teaching purposes in studies of radiolarian fossils, even in institutions where HF cannot be used owing to chemical restrictions.

## Introduction

The extraction of radiolarian fossils using hydrofluoric acid (HF) began in the 1970s^1,2^. The establishment of this technique, coupled with the increased use of scanning electron microscopy (SEM), resulted in a large increase in taxonomic and biostratigraphic studies of radiolarians, which have since become an essential reference fossil for the study of Earth’s history^3–6^. Radiolarian fossils have an important role in geological dating and in several other research fields, such as the identification of accretionary complexes^7,8^ and mass extinctions at the Permian–Triassic and Triassic–Jurassic boundaries^9–11^.

However, there are several major problems with studying radiolarian fossils using the HF method. First, HF is a highly reactive chemical. This makes it extremely harmful to the human body, and it is difficult to handle and store^12,13^. Therefore, the HF method requires the use of high-efficiency fume hoods and gas masks, as well as a system for the strict storage and management of HF^13,14^. In addition, the use and disposal of HF is severely restricted in most countries because of its significant effects on plant and soil ecosystems and hydrological systems^15^. Furthermore, the increasing demand for HF in a wide range of industries, such as fluoropolymers and the semiconductor industry^16,17^, has led to rising HF prices. For these reasons, microfossil research using the HF method is only carried out in a limited number of research institutions.

We have investigated alternative methods that would allow the extraction of radiolarians to be conducted more safely and easily. Radiolarian cherts (Supplementary Fig. S1) consist of biogenic particles, such as radiolarians and conodonts, in a matrix of microcrystalline and cryptocrystalline quartz and clay minerals. The extraction of radiolarians from such siliceous sedimentary rocks requires the efficient dissolution of the quartz matrix^2^. The dissolution of siliceous rocks using alkaline solutions (sodium or potassium hydroxide) has long been known, and alkaline treatment of radiolarians was attempted in Europe in the first half of the twentieth century^18,19^. However, since the establishment of the HF method, there have been only a few reports on the extraction of radiolarian fossils using alkaline solutions^20^. Choquette et al*.*^21^ conducted experiments on the solubility of various minerals and rocks in aqueous sodium hydroxide (NaOH) solutions. They found that in the case of chert, the alkaline solution on the surface of the chert matrix rapidly dissolved the microcrystalline quartz. Recently, Rigo et al*.*^22^ found that the solubility of chert in NaOH solutions increases markedly with increasing temperature. They reported the use of 3 mol/L NaOH at 80 °C to extract well-preserved conodonts from chert. Although most dissolution reactions of chert with NaOH do not occur at room temperature^21^, it may be possible to dissolve chert and obtain radiolarians at higher temperatures^21,22^.

We undertook NaOH dissolution experiments on Triassic radiolarian cherts under different temperature and concentration conditions. As a result, we succeeded in extracting radiolarian fossils using a low-concentration NaOH solution (1 mol/L). The radiolarian skeletons in the studied Triassic cherts are composed of microcrystalline quartz transformed from opal-A through opal-CT^23,24^. Although radiolarian opal dissolves well in NaOH solutions^25^, the dissolution rate of radiolarian skeletons composed of microcrystalline quartz is significantly slower than that of opal^21^. Therefore, we also present major element data and surface observations of the chert samples to assess why radiolarians can be extracted from cherts using NaOH.

### Chert dissolution experiment using NaOH

The chert samples used in the experiments were collected from the Upper Triassic Sections^10,26–30^ of the Mino Belt, central Japan (Supplementary Fig. S1). The results of thin section observation and X-ray powder diffraction (XRD) analysis (Supplementary Figs. S1 and S2) indicate that the radiolarians in the studied chert are composed of microcrystalline quartz. Dissolution experiments were first carried out on powdered chert samples to constrain the dissolution conditions in terms of temperature and NaOH concentration. We used samples of 4.0 g, 40 mL of 1–4 mol/L NaOH solution, temperatures of 60–100 °C, and a dissolution time of 1–5 h. After the dissolution experiments, the remaining sample was neutralised, dried, weighed, and subjected to X-ray fluorescence (XRF) spectrometry analysis to determine the nature of the dissolved elements and minerals (see the Methods).

**Figure 1:**
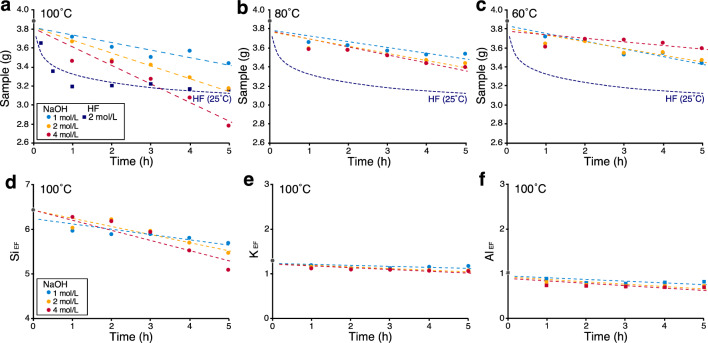
Chert dissolution experiments using the NaOH method. Powdered chert samples (4.0 g) were dissolved in 40 mL of NaOH at different concentrations (1, 2, and 4 mol/L) and temperatures (60, 80, and 100 °C). Grey circles at 0 h show the results for a sample of 4.0 g of chert powder added to 40 mL of ultrapure water, which was centrifuged and dried for comparison. Sample KTY-220 from the Katsuyama section (upper Rhaetian) was used in the experiment. See Supplementary Tables S1 and Table S2 for data on this figure.

When the chert samples were dissolved in NaOH at 100 °C, substantial weight loss was observed at all concentrations (Fig. 1a). The decrease was greater at higher concentrations. After 5 h, the weight loss was ~ 1.0 g in the 4 mol/L solution compared with ~ 0.2 g in the 1 mol/L solution. However, experiments at 60 and 80 °C showed that the solubility was lower than that at 100 °C, regardless of the NaOH concentration (Fig. 1b–c). This study compared dissolution rates in HF (2 mol/L; 25 °C; 24 h) and NaOH. For the HF method, we observed a decrease in weight (~ 0.8 g) and reached chemical equilibrium within 1 h (Fig. 1a), as expected from the equilibrium constant (3.4 × 10^6^ at 25 °C for SiO_2_ + 6HF  ⇄  H_2_SiF_6_ + 2H_2_O)^31^. In contrast, the NaOH method required approximately 3 h at 4 mol/L and 15 h at 1 mol/L to dissolve the same amount of sample (0.8 g) as the HF method, which is indicative of constant and gradual dissolution of chert in the NaOH.

The XRF analyses showed that the cherts were variably affected by NaOH dissolution in terms of their mineral components. The chert samples consist mainly of quartz and clay minerals (illite and chlorite; Supplementary Fig. S1e), with minor apatite (conodont) and other minerals (hematite, magnetite, and rutile^32,33^). Dissolution of the characteristic elements of each mineral was evaluated using enrichment factors (EFs; see the Methods). This is an index of concentration variation relative to an element unaffected by dissolution in NaOH (i.e., Ti). The XRF results show that the absolute contents of Si decreased with time (Fig. 1d). The Al and K, which occur mainly in clay minerals (e.g., illite), decreased slightly as the dissolution experiment progressed (Fig. 1e–f). These data suggest that the NaOH solution mainly dissolved quartz, because the dissolution of clay minerals was less than that of quartz.

**Figure 2:**
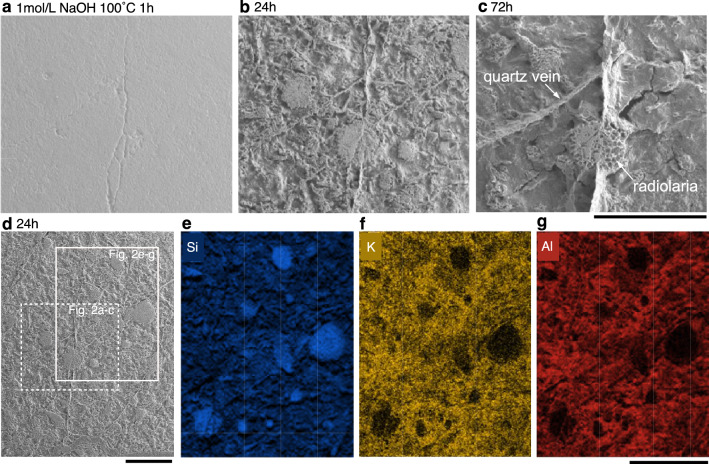
Temporal changes in the chert surface during NaOH dissolution. (**a**–**d**) Changes in the chert surface after dissolution in 1 mol/L NaOH at 100 °C for 1, 24, and 72 h. Note the preferential dissolution of the matrix and appearance of radiolarians. (**e**–**g**) Chemical maps (Si, K, and Al) of the chert surface obtained by SEM–EDS analysis after 24 h of NaOH dissolution. Sample KTY-76 from the Katsuyama section (upper Rhaetian) was used in this experiment. Scale bars = 500 µm.

Since the experiments showed that even a NaOH solution with a concentration of 1 mol/L could dissolve chert at 100 °C, we used this solution to observe the progress of dissolution of the chert surface. SEM observations showed that dissolution occurred mainly in the chert matrix, with the radiolarians and quartz veins being less affected by dissolution (Fig. 2). SEM–energy dispersive X-ray spectroscopy (EDS) analysis indicated that the chert matrix is a mixture of cryptocrystalline quartz and illite, with the latter being rich in K and Al. Therefore, the alkaline aqueous solution on the surface of the matrix rapidly dissolves the cryptocrystalline quartz, which is indicative of complete dissolution of the chert samples, as suggested in a previous study^21^.

### Comparison of the NaOH and HF methods

**Figure 3:**
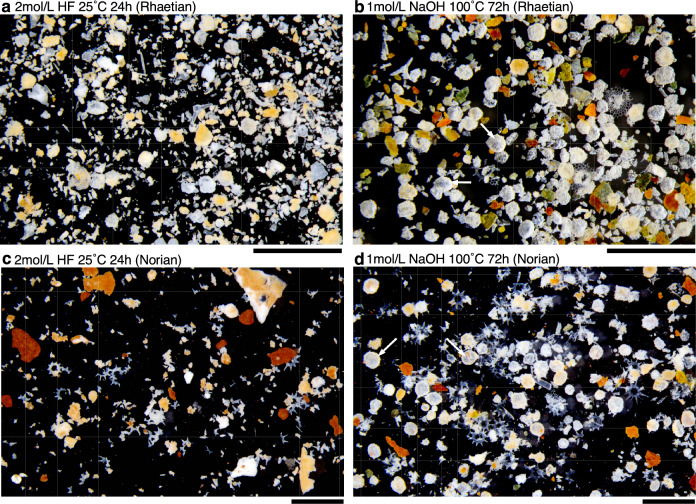
Comparison of residues after the NaOH and HF methods. The NaOH method preserves spherical Spumellaria (arrow) more successfully than the HF method. Radiolarian skeletons extracted using the NaOH method are less corroded and more transparent than those extracted using the HF method. (**a**–**b**) Sample KTY-76 from the Katsuyama section (upper Rhaetian). (**c**–**d**) Sample NHR-181 from the Sakahogi section (upper Norian). Scale bars = 1 mm.

The dissolution experiments were carried out on 5.0 g of 4–8 mm-sized chert fragments, and the residue was compared with that obtained from the conventional HF method (2 mol/L; 25 °C; 24 h). The amount of radiolarians obtained from the same sample was markedly higher after using the NaOH method (Fig. 3). This is particularly evident for the spherical group of Spumellaria. Even if the spumellarians are preserved by the HF method, their surface of the skeleton is corroded by the HF and appears whitish under a stereomicroscope (Fig. 3). These differences may be related to the rapid dissolution rate of the HF method. Presumably, the HF attacks the sample surface very quickly (Fig. 1a), and the difference in the dissolution rate between the radiolarians and the chert matrix is not as pronounced for the HF method compared with the NaOH method.

**Figure 4:**
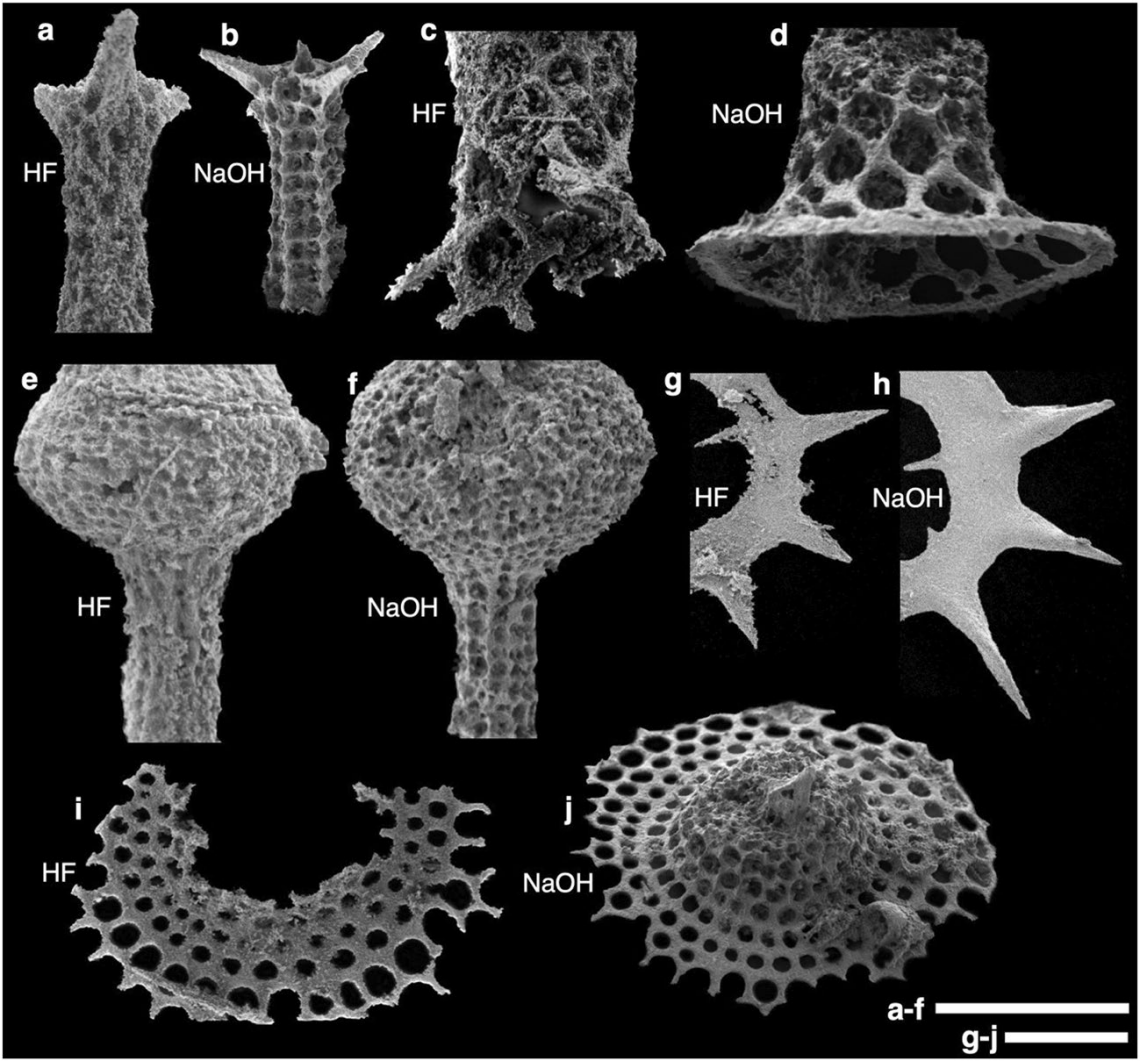
Preservation of radiolarian skeletons after the HF and NaOH extraction methods. The best-preserved radiolarians from the same sample were compared. A full image of each radiolarian fossil is shown in Supplementary Fig. S3. See also a report on these radiolarians extracted from the same Section 10 using the HF method for comparison. Scale bars = 100 µm. (**a**–**b**) Pseudohagiastrum giganteum Carter and Hori from sample KTY-220 from the Katsuyama section (upper Rhaetian). The NaOH-extracted specimen clearly shows that the meshwork is latticed and linearly arranged, which is characteristic of P. giganteum. (**c**–**d**) Deflandrecyrtium ithacathum (Sugiyama) from sample KTY-220. A specimen preserved to the edge of the skirt obtained by the HF method could not be found in the present study. (**e**–**f**) Globolaxtorum tozeri Carter from sample KTY-220. The NaOH-extracted specimen preserves square to rectangular pore frames on a terminal tube. G. tozeri extracted using the NaOH method also retains short downward sloping thorns at the tip of the tube (Supplementary Fig. S3a), which were not observed in the HF method. (**g**–**h**) Praemesosaturnalis rugosus (Yeh) from sample NHR-181 from the Sakahogi section (upper Norian). The HF-extracted specimen shows saturnian ring corrosion, making it difficult to observe the loose twisting of the external rays that is characteristic of P. rugosus. (**i**) Skirt F Sugiyama, sample KTY-76 from Katsuyama section (upper Rhaetian). (**j**) Deflandrecyrtium takemurai (Yeh and Cheng), sample KTY-76. Although the HF-extracted specimen (**i**) has four rows of subcircular pores on the skirt, the cephalis and thorax parts are poorly preserved and are thus tentatively referred to as the Skirt F34. However, the NaOH method preserves the cephalis and thorax, indicating it belongs to the genus Deflandrecyrtium Kozur and Mostler. The rows of pores are also present in the NaOH-extracted specimen from the skirt to thorax, and the cephalis also has an apical spine. These characteristics are not well preserved by the HF method (Supplementary Fig. S3).

Observations of the surface of the skeletons by SEM clearly show that the NaOH method better preserves radiolarians compared with the HF method (Fig. 4). For example, comparing the late Norian (Upper Triassic) species, the HF method corrodes the tip of the spine and thin rings of the shell (Fig. 4g), whereas these areas are well preserved after the NaOH method. In particular, twisting of the tip of the spine, which was difficult to observe with the HF method, can be observed after the NaOH method (Fig. 4h). The NaOH method also tends to preserve thin skeletons particularly well, such as the skirts of Rhaetian nasellarians (Fig. 4d), which are poorly preserved using the HF method. In addition, radiolarians that could not be identified to species level owing to poor preservation and had been referred to by partial character names (e.g., Skirt F^34^) could be observed in their entirety using the NaOH method (Fig. 4j).

### Recommended method and its significance

This study has revealed that radiolarian quartz skeletons dissolve less rapidly in NaOH than the cryptocrystalline quartz matrix, and that the newly proposed NaOH technique can be used to efficiently recover radiolarian fossils from chert. The amount of chert dissolution is strongly dependent on temperature. Although the amount of dissolution is low at < 80 °C, when heated to 100 °C, even a 1 mol/L NaOH solution can dissolve chert and extract radiolarian fossils.

NaOH with a concentration of < 1 mol/L is classified one category lower than > 2 mol/L NaOH in the United Nations Globally Harmonized System of Classification (UN-GHS) of hazardous chemicals for skin and respiratory hazards^35^. As such, a 1 mol/L NaOH solution is easy to handle and has little effect on human health. Furthermore, NaOH is less expensive than HF. Accordingly, the NaOH method is a much safer and more efficient method of extracting radiolarians than the conventional HF method.

**Figure 5:**
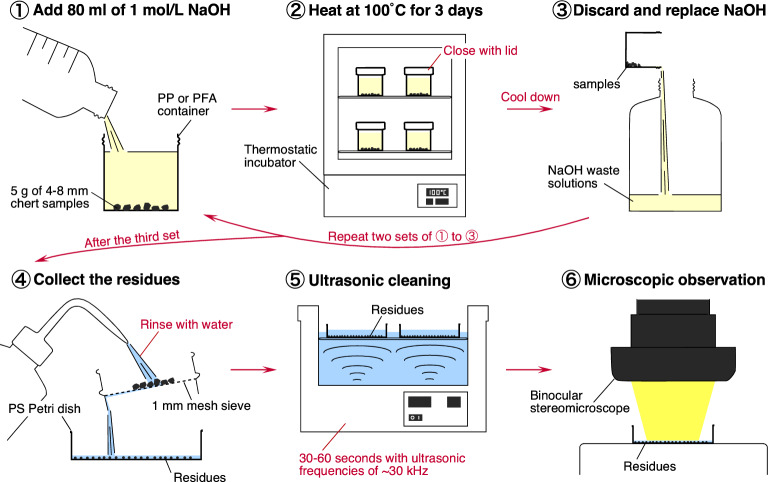
A newly proposed method for extracting radiolarians using dilute NaOH. Steps ① to ③ should be repeated five to six times to dissolve the chert sample. The collection of residues in step ④ is best started after the third repeat.

In summary, we propose a new method using 1 mol/L NaOH for the extraction of radiolarian fossils (Fig. 5). Radiolarians can also be extracted from chert using 3 mol/L NaOH^22^, which has the advantage of dissolving the chert more quickly (Fig. 1a). However, due to the high rate of dissolution, there are many large fragments of chert in the residue, which make it difficult to separate the radiolarians. For this reason, we recommend the use of 1 mol/L NaOH as described below.

**Figure 6:**
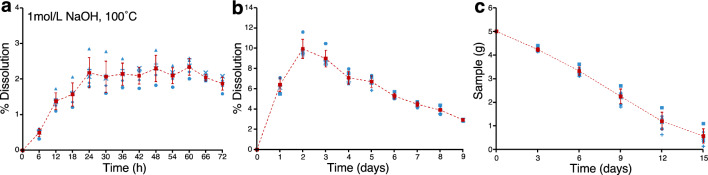
Dissolution results for the chert fragments. (**a**–**b**) Percentage dissolution between each time step when 5.0 g of 4–8 mm-sized chert pieces (KTY-220; Katsuyama section) were dissolved in 80 mL of 1 mol/L NaOH at 100 °C. Weight loss at each time step is calculated relative to the original weight (~ 5 g). The blue symbols show the measured results, and the red symbols show the mean and standard deviation of measurements at each time. The dissolution rate peaks at 2–3 day and then decreases. (**c**) Weight loss after changing the NaOH solution every 3 day under the same conditions as in (**a**–**b**). All 5.0 g of a chert sample can be dissolved by changing the NaOH around five to six times every 3 day. See Supplementary Table S3 for data on this figure.

First, 5 g of 4–8 mm-sized chert fragments containing radiolarians should be placed in a 100 mL polypropylene (PP; heat resistant temperature of ~ 120 °C) or perfluoroalkoxy alkane (PFA; heat resistant temperature of ~ 250 °C) sample container. Subsequently, 80 mL of 1 mol/L NaOH solution should be added, and the sample covered and left to react for 3 d at 100 °C in a thermostatic incubator. The NaOH solution should then be replaced with a fresh NaOH solution. The reason for this is that the dissolution reaction proceeds at a constant rate for about 3 day and then slows down after 3–5 days (Fig. 6a–b). Note that it is dangerous to open the lid immediately after heating, and it is best to allow the solution to cool before replacing the NaOH. Repeating this step five to six times will dissolve all of the chert sample (Fig. 6c). Given that the first two dissolutions are not sufficient to dissolve material from which the radiolarians are extracted (Fig. 2c), the residues from these do not need to be collected.

After the third dissolution step, the residue can be collected in a polystyrene (PS) Petri dish. Ultrasonic cleaning of the recovered residue pulverises the clay minerals and cryptocrystalline quartz aggregates that form the chert matrix. This makes the radiolarians much easier to observe (Supplementary Fig. S4). Ultrasonic frequencies of ~ 30 kHz do not produce enough cavitation to destroy the radiolarians.

Microfossil extraction using the low-concentration NaOH method is superior to the HF method in three ways: (1) it has little effect on the human body and environment; (2) it allows easy chemical management (i.e., storage and liquid waste treatment); and (3) it extracts fossils with better preservation and primary chemical compositions. (1) and (2) allow the extraction of radiolarian fossils in institutes and schools where handling of microfossils was not previously possible due to equipment and chemical regulations. This will further accelerate research in the fields of radiolarian biostratigraphy and the geological timescale, which increased in the 1980s and 1990s^4–6^. Point (3) will provide new information on previously overlooked morphological features of radiolarian fossils. For example, some morphological characteristics, such as Skirt and Spine^28^, which could not be identified to species level owing to poor preservation, have been used in Japanese radiolarian biostratigraphy^36^. The NaOH method preserves a more complete skeletal structure associated with these characteristics, which had not been previously possible (Fig. 4i). Because of these advantages, we plan to further test the NaOH method on siliceous rocks other than radiolarian chert (e.g., spicular and diatom cherts) and on chert samples containing calcified radiolarians. The NaOH method not only increases the accessibility of microfossil research but is also expected to provide new insights into biostratigraphy, fossil assemblages, taxonomy, taphonomy, and microfossil morphological analyses.

Furthermore, the NaOH method is effective in extracting conodont fossils from cherts^22^. Conodonts were extracted from cherts even at the low concentrations (1 mol/L) of NaOH proposed in this study. Moreover, unlike HF, the NaOH method does not affect the chemical composition of apatite^22^, which is the main component of conodonts. By obtaining well-preserved conodonts that retain their original chemical composition using the NaOH method, it will be possible in the future to analyse the O isotopes of apatite^37,38^ and reconstruct the Rare Earth Element (REE) composition of palaeo-seawater^39^. Similarly, although fluoride formation may be a problem with the HF method, Si and O isotope analysis^40^ on individual radiolarians extracted using the NaOH method may be possible in the future. These isotopic analyses would provide new insights not only into paleoenvironmental studies, but also into silica phase transformations of radiolarian fossils during diagenesis^23^.

## Methods

### Dissolution of the chert samples

We collected an uppermost Triassic chert sample from the Katsuyama Section^10,26,29^ (Supplementary Fig. S1) for the dissolution experiments with NaOH. Each sample was crushed to a size of 1–4 mm and washed by ultrasonic cleaning in Milli-Q deionised water (> 18 MΩ). After drying, the fragments were pulverised using a Multi-Beads Shocker (PV1001; Yasui Kikai, Japan).

The effects of the NaOH solution on the cherts were investigated using three different concentrations (1, 2, and 4 mol/L) and temperatures (60, 80, and 100 °C) with five different reaction times (1, 2, 3, 4, and 5 h). To dissolve the samples, 4.0 g of sample powder and 40.0 mL of NaOH solution (1, 2, and 4 mol/L) were placed in 45 Teflon centrifuge tubes. A reference sample containing 40.0 mL of Milli-Q deionised water was also prepared for comparison. The centrifuge tubes were then placed in a thermostatic incubator and heated to 60, 80, or 100 °C for a set time. The NaOH was then removed from the centrifuge tube. The residues were rinsed seven times with 50 mL of Milli-Q water, centrifuged (3000 rpm; 3 min), and dried at 60 °C. The dried residues were homogenised in an agate mortar.

### Major element analysis

After drying the powdered samples at 60 °C, the samples were pressed at 2 × 10^4^ kg for 3 min to yield pressed powder pellets. The pellets were prepared from a mixture of cellulose solidifier (PANalytical) and the sample in a ratio of 1:5. Major element concentrations were determined using X-ray fluorescence (XRF) spectrometry (PANalytical Epsilon 3XLE with a Mo X-ray tube) on the pressed powder pellets at Kyushu University, Japan. Samples were calibrated using 20 standard rock samples issued by the Geological Survey of Japan. The accuracy of the calibration curve at the time of measurement was ± 0.08 wt.% Na_2_O, ± 0.05 wt.% MgO, ± 0.14 wt.% Al_2_O_3_, ± 0.56 wt.% SiO_2_, ± 0.03 wt.% P_2_O_5_, ± 0.06 wt.% K_2_O, ± 0.08 wt.% CaO, ± 0.03 wt.% TiO_2_, ± 0.01 wt.% MnO, and ± 0.19 wt.% Fe_2_O_3_. The reproducibility based on repeated analyses of four standards (JCh-1) was better than ± 0.5% for Mg, Al, Si, K, Ca, Ti, and Fe, and better than ± 1% for Na and Mn.

The major element data obtained via XRF analysis are compositional data with a sum of 100 wt.%, which means that if one element decreases in concentration, the other elements increase^41^. Therefore, to monitor the leaching of elements during NaOH dissolution, it is necessary to examine the relative variation of elements with respect to Ti contained in rutile, which is insoluble in NaOH. This study used the enrichment factor method^42–44^ to examine the variations in elemental concentrations. The data were normalised to Ti contents and compared with those of upper continental crust (UCC)^45^ to obtain enrichment factors as follows:

X_EF_ = (X_sample_/Ti_sample_)/(X_UCC_/Ti_UCC_)

where X and Ti are the weight contents of elements X and Ti, respectively.

### SEM–EDS observations

We selected chert samples from Upper Triassic chert sections in the Jurassic accretionary complexes from the Mino Belt in central Japan. Radiolarians were extracted from cherts of the Rhaetian Katsuyama Section^10,26,29^ (samples KTY-76, 220) and the Norian Sakahogi Section^27,28,30^ (NHR-181). The preservation of radiolarians (Fig. 4) extracted from the cherts using the HF method (2 mol/L; 25 °C; 24 h) and the NaOH method (1 mol/L; 100 °C; 15 day) was compared using a scanning electron microscope (SEM; HITACHI TM3030 Plus) at Kyushu University, Fukuoka, Japan. The elemental compositions of the observed cherts (Fig. 2) were determined by energy dispersive X-ray spectroscopy (EDS; Bruker Quantax at Kyushu University) with an accelerating voltage of 15 kV.

### Supplementary Information

Supplementary Information.

Supplementary Table S1.

Supplementary Table S2.

Supplementary Table S3.

## Data Availability

All data generated or analyzed during this study are included in this published article and supplementary information files.
