# Supplementary material for: A dilute sodium hydroxide technique for radiolarian extraction from cherts
Source: Sci Rep. 2024 Jun 17;14:12831. doi: 10.1038/s41598-024-63755-9 (PMC11183093; doi:10.1038/s41598-024-63755-9)
Supplement: Supplementary file 1 — Supplementary Information. [file 41598_2024_63755_MOESM1_ESM.docx]

**Supplementary Figures of ‘A dilute sodium hydroxide technique for radiolarian extraction from cherts’ by Onoue et al.**

**Supplementary Fig. S1.** Location of study sections and samples used in this study. **a,** Geological map showing the location of the study area in the Mino Belt, central Japan^1^. **b,** Route map showing the locality of the study sections along the middle reaches of Kiso River^2^. These maps are created using ACD Systems Canvas Draw software (Version 6.0) (<https://www.poladigital.co.jp/canvas/index.html>). **c,** Columnar sections of Katsuyama^2,3^ and Sakahogi^4,5^ sections showing the stratigraphic levels of samples used in this study. **d,** Photograph of an outcrop of the lower Rhaetian bedded chert in the Katsuyama section. **e,** X-ray powder diffraction (XRD) patterns of the chert from the Katsuyama section (sample KTY-220). XRD patterns were obtained using Rigaku Mini Flex-II X-Ray diffractometer equipped at Kyushu University with a tube acceleration voltage and current of 30 kV and 15 mA, respectively. XRD analyses were conducted from 3° to 90° 2-theta with a step size of 0.01°.


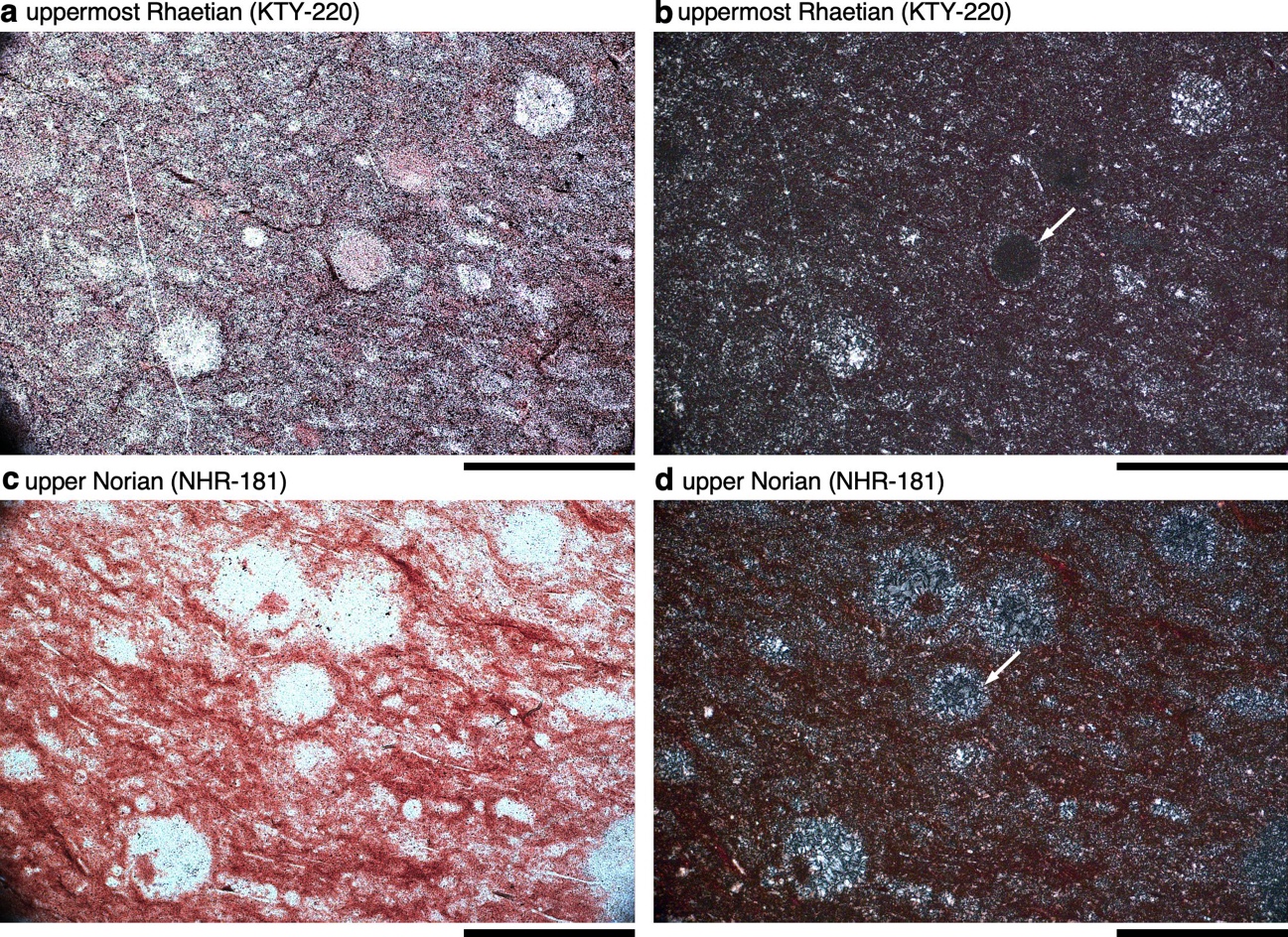


**Supplementary Fig. S2.** Thin-section photomicrographs of chert samples with radiolarians (arrow) from the (**a, b**) uppermost Rhaetian (sample KTY-220) and **(c, d**) upper Norian (sample NHR-181) from Katsuyama and Sakahogi sections. Plane (**a, c**) and cross (**b, d**) polarized light. Scale bars = 500 µm.


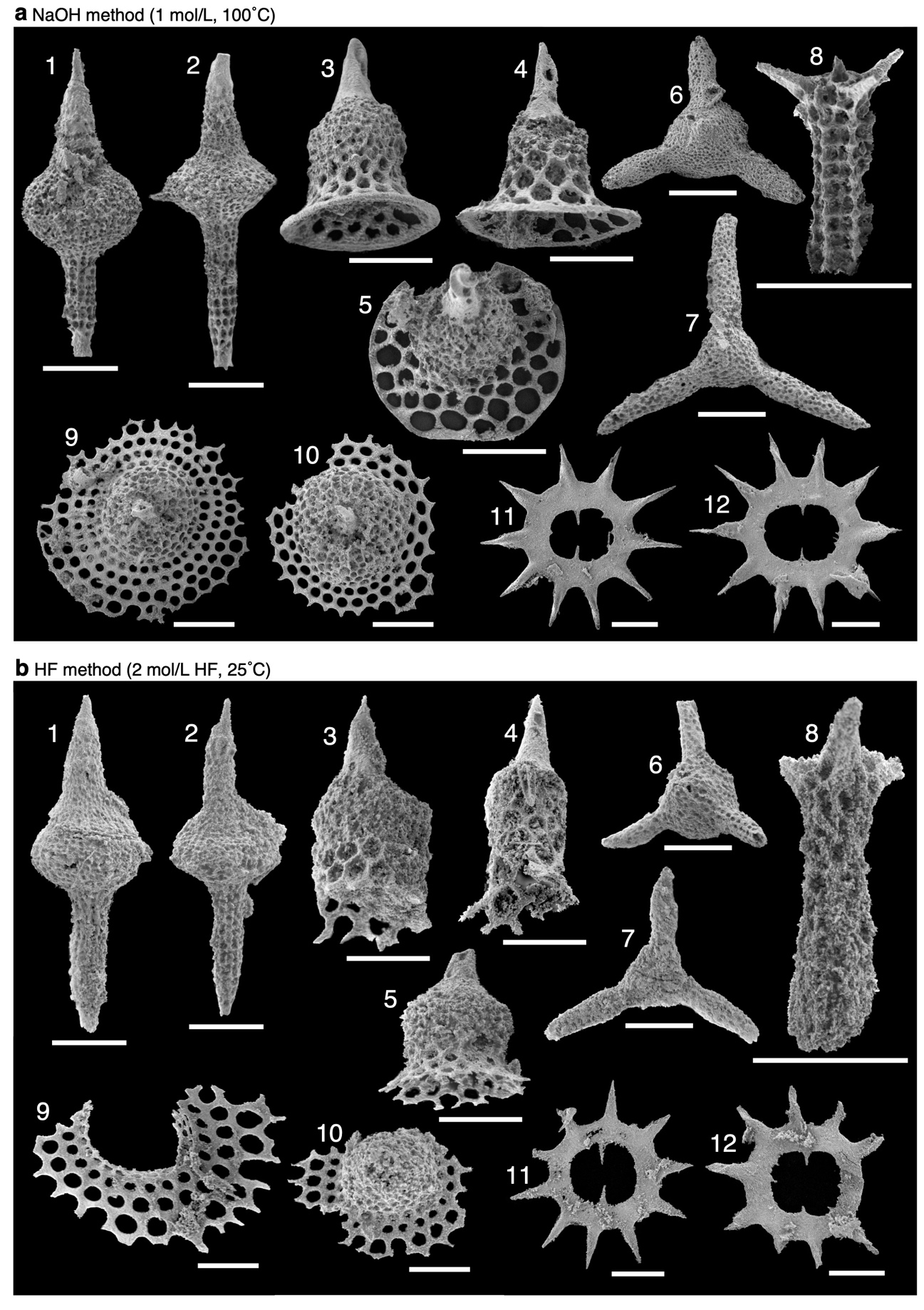


**Supplementary Fig. S3 (Caption on next page)**

**Supplementary Fig. S3.** Preservation of radiolarians extracted using NaOH and HF methods. **a,** Characteristic radiolarians from Katsuyama and Sakahogi sections obtained by the NaOH method (1 mol/L, 100˚C, 15 days). 1–10 are latest Rhaetian, 11–12 are late Norian (Sevatian). 1–2, *Globolaxtorum tozeri* Carter, Sample KTY-220. 3–4, *Deflandrecyrtium ithacathum* (Sugiyama), KYT-220. 5, *Deflandrecyrtium carterae* Yeh and Cheng, KYT-220. 6, *Livarella valida* Yoshida, KTY-220. 7, *Livarella gifuensis* Yoshida, KTY-220. 8, *Pseudohagiastrum giganteum* Carter and Hori*,* KTY-220. 9–10, *Deflandrecyrtium takemurai* (Yeh and Cheng), KTY-76. 11–12, *Praemesosaturnalis rugosus* (Yeh), NHR-181. **b,** Characteristic radiolarians from Katsuyama and Sakahogi sections obtained by the HF method (2 mol/L, 25˚C, 24 hours). 1–10 are latest Rhaetian, 11–12 are late Norian (Sevatian). 1–2, *Globolaxtorum tozeri* Carter, Sample KTY-220. 3–4, *Deflandrecyrtium* sp. *D.* cf. *ithacathum* (Sugiyama), KYT-220. 5, *Deflandrecyrtium* sp., KYT-76. 6, *Livarella valida* Yoshida, KTY-220. 7, *Livarella* sp. *L.* cf. *gifuensis* Yoshida, KTY-220. 8, *Pseudohagiastrum* sp. *P.* cf. *giganteum* Carter and Hori*,* KTY-220. 9, Skirt F Sugiyama, KTY-76. 10, *Deflandrecyrtium* sp. *D.* cf. *takemurai* (Yeh and Cheng) KTY-76. 11–12, *Praemesosaturnalis rugosus* (Yeh), NHR-181.


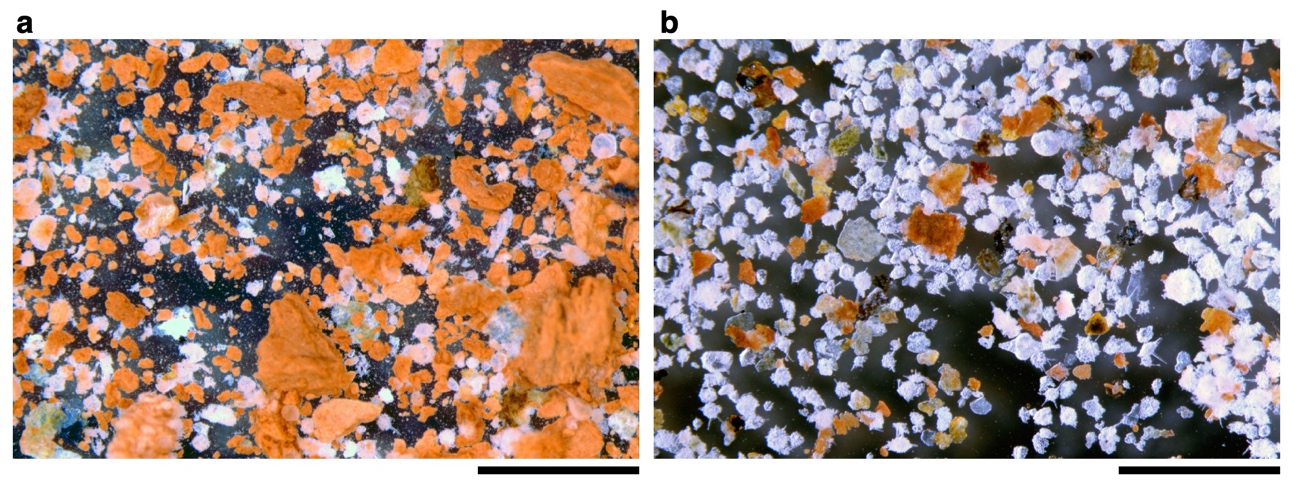


**Supplementary Fig. S4.** Chert residues before (a) and after (b) ultrasonic cleaning. Ultrasonic cleaning effectively pulverises the aggregates of clay minerals and crypto-crystalline quartz that form the matrix of the chert. Scale bars = 1 mm. Sample KTY-254 from Katsuyama section.

**References**

1 Onoue, T. et al. Bolide impact triggered the Late Triassic extinction event in equatorial Panthalassa. *Sci. Rep.* **6**, 29609 (2016).

2 Hori, R. Radiolarian biostratigraphy at the Triassic/Jurassic period boundary in bedded cherts from the Inuyama area, central Japan. *J. Geosci. Osaka City Univ.* **35**, 53–65 (1990).

3 Du, Y. et al. The asynchronous disappearance of conodonts: new constraints from Triassic-Jurassic boundary sections in the Tethys and Panthalassa. *Earth-Sci. Rev.* **203**, 103176 (2020).

4 Uno, K., Yamashita, D., Onoue, T. & Uehara, D. Paleomagnetism of Triassic bedded chert from Japan for determining the age of an impact ejecta layer deposited on peri-equatorial latitudes of the paleo-Pacific Ocean: A preliminary analysis. *Phys. Earth Planet. Inter.* **249**, 59–67 (2015).

5 Sato, H. et al. Rhenium-osmium isotope evidence for the onset of volcanism in the central Panthalassa Ocean during the Norian “chaotic carbon episode”. *Glob. Planet. Change* **229**, 104239 (2023).
